# Amp-PCR: Combining a Random Unbiased Phi29-Amplification with a Specific Real-Time PCR, Performed in One Tube to Increase PCR Sensitivity

**DOI:** 10.1371/journal.pone.0015719

**Published:** 2010-12-31

**Authors:** Lena Erlandsson, Lars Peter Nielsen, Anders Fomsgaard

**Affiliations:** 1 Virus Research and Development, Department of Virology, Statens Serum Institut, Copenhagen, Denmark; 2 Influenza Laboratory, Department of Virology, Statens Serum Institut, Copenhagen, Denmark; New England Biolabs, Inc., United States of America

## Abstract

In clinical situations where a diagnostic real-time PCR assay is not sensitive enough, leading to low or falsely negative results, or where detection earlier in a disease progression would benefit the patient, an unbiased pre-amplification prior to the real-time PCR could be beneficial. In Amp-PCR, an unbiased random Phi29 pre-amplification is combined with a specific real-time PCR reaction. The two reactions are separated physically by a wax-layer (AmpliWax®) and are run in sequel in the same sealed tube. Amp-PCR can increase the specific PCR signal at least 100×10^6^-fold and make it possible to detect positive samples normally under the detection limit of the specific real-time PCR. The risk of contamination is eliminated and Amp-PCR could replace nested-PCR in situations where increased sensitivity is needed e.g. in routine PCR diagnostic analysis. We show Amp-PCR to work on clinical samples containing circular and linear viral dsDNA genomes, but can work well on DNA of any origin, both from non-cellular (virus) and cellular sources (bacteria, archae, eukaryotes).

## Introduction

Clinical laboratories around the world commonly uses specific real-time PCR [1] for virus identification which is a rapid, sensitive and specific method that is easy to perform, but has certain limitations. The sensitivity and specificity of any given virus-specific PCR vary depending on the robustness of the primers and the genome variability or mutations in the primer-binding region of the viral genome, as well as the target copy number and the efficiency of the reaction. Sometimes the PCR is not sensitive enough to detect the causing agent in clinical situations with expected low viral load or limited amount of sample, even if every measure has been taken to design the most sensitive primers and probe from the sequence known about a specific virus. Moreover, there appears to be an inherent limitation to the PCR amplification of small amounts from complex samples, known as the “Monte Carlo effect” [2]. Complex samples, such as a clinical sample, containing a low viral copy number close to the detection level of a PCR assay will experience large variations in amplification and reduced reproducibility. These limitations of the PCR technique can lead to low or falsely negative results, which in turn may have consequences for the diagnosis and treatment of patients. For example, in a multi-laboratory study of Herpes Simplex virus PCR, which is a widely used and validated PCR assay for cerebrospinal fluids (CSF), it was found that low-level positives were often missed [3].

One current technique used to increase the sensitivity of a virus-specific PCR-assay is nested-PCR [4], where two sets of primers are used in two successive PCR-runs. The second set of primers, are intended to amplify a target within the amplicon produced in the first run by the first set of primers. However, the transfer of PCR amplicons from the first to the second run, present a serious risk of contamination in a routine laboratory, and the need for two PCR reactions makes is more time consuming.

Random whole genome amplification (WGA) by isothermal amplification using the Phi29 DNA polymerase has established itself as an excellent alternative to random-PCR based amplification. By isothermal multiple displacement amplification (MDA) in the presence of random primers, Phi29 DNA polymerase allows for uniform amplification across genomes with less than 3-fold bias [5], [6], [7]. Furthermore, the Phi29 DNA polymerase has the highest processivity rate reported, ∼70 000 bases every time it binds [8] and high fidelity with an error rate of only 1 in 10^6^–10^7^ bases [9].

Here we report about a method we call Amp-PCR that, in one tube, combines random unbiased isothermal Phi29 amplification with a virus-specific real-time PCR which increases the PCR signal at least 100×10^6^-fold and makes it possible to detect amounts of sample normally under the detection limit of a specific PCR. Since both assays are performed after each other in the same closed tube we eliminate the risk for contamination as no transfer of pre-amplified sample is needed. We show this to work on clinical samples containing both circular and linear double-stranded viral DNA genomes, but the described method would work on any DNA of any origin, as well as on cDNA of sizes 2000 bp or larger.

## Materials and Methods

### Ethics statement

This study was an improvement of the routine diagnostic assay used at the Department of Virology, Statens Serum Institut, Copenhagen, Denmark. A statement given by the Danish ethics committee of Copenhagen (Videnskabsetiske Komiteer for Region Hovedstad), as a response to our enquiry, declare that according to Danish law (Sundhedsloven) clinical samples received for routine diagnostic analysis at Statens Serum Institut, Copenhagen, Denmark (accredited and quality-controlled Danish National reference diagnostic laboratory (ISO 17025); www.ssi.dk) can be used for quality control, assay development and improvement of routine techniques without ethics committee approval or written consent from the patient. It was a blinded study with no patient contact or patient information, only sample information.

### Virus samples

Clinical samples used for this study were two JC polyomavirus (JCV)-positive CSF samples, one human papillomavirus type 16 (HPV16)-positive cervical smear and one human herpes simplex-1 virus (HSV1)-positive swab sample. The JCV and the HPV16 are both circular double-stranded DNA viruses of 5.1 and 7.9 kb, respectively. HSV1 virus is a linear double-stranded DNA virus of 152 kb.

### Pre-treatment and extraction of viral DNA in clinical samples

Two hundred microliters of clinical sample was transferred to an eppendorf tube and centrifuged at 17000 g for 10 minutes to remove cell debris. The supernatant was then filtered through a 0.22 µm Ultrafree MC spin filter (Millipore) at 2000 g for 2 minutes and thereafter DNase treated (6 U DNaseI Amplification grade, Invitrogen) for 1½ hours at room temperature while shaken in a Thermomixer (AH Diagnostics). The viral DNA was extracted using the PureLink Viral RNA/DNA kit (Invitrogen), without the addition of carrier RNA. The extracted viral DNA was eluted with 20–30 µl DNase/RNase-free sterile water, and stored at −20°C or immediately used.

### Virus-specific real-time PCR

Real-time PCR master mixes specific for JCV, HPV16 or HSV1 were prepared, using described primers and probe for JCV [10], [11] and in-house assays for HPV16 (primers HPV16-E6-12F (5′- CGA CCC AGA AAG TTA CCA CAG TT-3′) and HPV16-E6-12R (5′- TGT TGC TTG CAG TAC ACA CAT TCT A-3′), and probe HPV16-E6-12P (5′-FAM- CAC AGA GCT GCA AAC AAC TAT ACA TGA TAT AAT-BHQ1-3′)) and HSV1 (primers HSV1-LPs (5′-TGT GGT GTT TTT GGC ATC AT-3′) and HSV1-LPas (5′- CCG ACA AGA ACC AAA AGG AA -3′), and probe HSV1-LPprobe (5′-FAM- CAT GCG TGC CGT TGT TCC CA-BHQ1-3′)). Thirty microliters of virus-specific master mix was prepared containing 15 µl of 2x QuantiTect Multiplex PCR NoROX Master mix (contains HotStarTaq DNA polymerase, Qiagen), 500 nM of each primer and 100 nM probe.

### Repli-g amplification and virus-specific PCR combined in Amp-PCR

**Figure 1:**
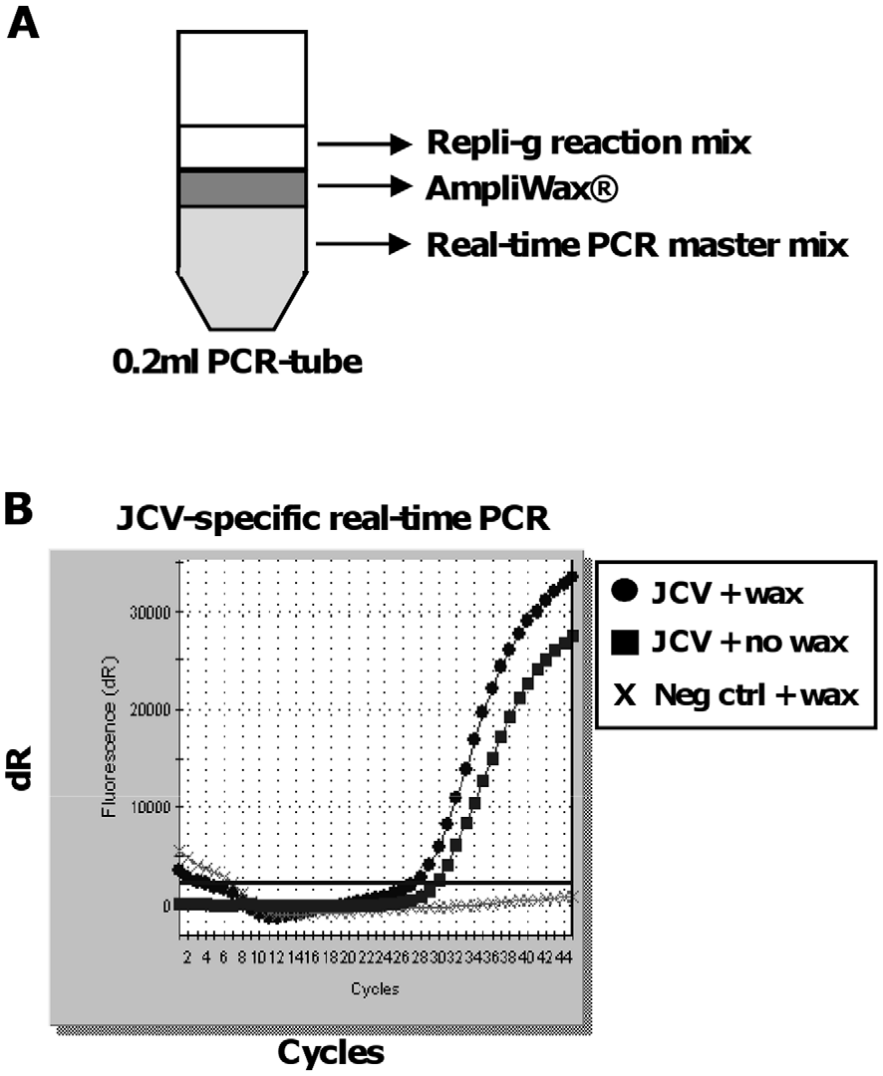
Description of the Amp-PCR method. In Amp-PCR a random pre-amplification step is combined with a specific real-time PCR and the two reactions performed in sequel in the same closed tube. A physical barrier is created between the two reactions by the use of an AmpliWax®-layer. The Phi29-based pre-amplification (Repli-g) is performed first at 30°C, followed by 95°C to inactivate Phi29 plus activate the Taq enzyme and to melt the wax, and finally the specific real-time PCR is performed. (A) Schematic picture of the Amp-PCR reaction in a 0.2 ml PCR-tube, including the Repli-g reaction, the AmpliWax®-seal and the specific real-time PCR reaction. (B) JCV-specific real-time PCR with or without AmpliWax®, which do not interfere with the JCV-specific PCR reaction. Negative control is water.

Briefly, in Amp-PCR, a random unbiased isothermal Phi29 amplification (Repli-g Midi, Qiagen) reaction was combined with a virus-specific real-time PCR and the two reactions physically separated by a gem of AmpliWax® melted in-between the two layers (Figure 1A). In this way, the Repli-g reaction could be performed first, at 30°C, while separated from the PCR master mix. Furthermore, the HotStarTaq polymerase present in the QuantiTect Multiplex PCR buffer in the PCR master mix is inactive at 30°C. After completed Repli-g reaction, 15–30 minutes incubation at 95°C inactivated the Phi29 DNA polymerase, activated the HotStarTaq polymerase and melted the wax so that the two reactions, the Repli-g and the PCR master mix, could be mixed. Thereafter the PCR reaction was run for 45 cycles using the amplified DNA as template.

In detail, thirty microliters of virus-specific master mix was put in a 0.2 ml PCR-micro tube. One pellet of AmpliWax® PCR Gem 50 (PE Applied Biosystems) was added on top of the PCR master mix, the tube put in a cycler and incubated at 60°C for 5 minutes to melt the wax followed by cooling at 37°C to solidify the wax on top of the PCR master mix. Next, the random Phi29 amplification reaction was prepared by making a 10 µl Repli-g Midi reaction (1/5 of the normal reaction) according to manufacturer's protocol (Repli-g Midi kit, Qiagen). Briefly, 1 µl viral DNA was mixed with 1 µl of denaturation solution and incubated at room temperature for 3 minutes. Two microliters of stop solution was added and the sample mixed. Thereafter, 5.8 µl of Repli-g Midi reaction buffer and 0.2 µl of Phi29 DNA polymerase was added and the sample vortexed. After a short centrifugation, the Repli-g reaction was added on top of the solidified AmpliWax®, the tube closed and the assay performed in a real-time Mx3005P-cycler (Stratagene). A PCR control (called PCR only) was always included by adding 1 µl viral DNA sample in 9 µl water on top of a wax-sealed master mix. The following program was run: 30°C for 16 hours to run the Repli-g reaction; 95°C for 15–30 minutes to inactivate the Phi29 DNA polymerase, to activate the Taq polymerase and to melt the wax so that the Repli-g product was mixed with the PCR master mix; followed by a 45 cycle-real-time PCR with 95°C for 15 seconds and 60°C for 1 minute.

### Positive control for JCV

Standard curves to determine JCV copy number in clinical samples were set up using the JCV Mad-1 pM1TC, kindly provided by Dr Eugene Major at NINDS, NIH, Bethesda, USA [11], at 10-fold dilutions starting at 1.8×10^7^ copies/µl (100 pg) down to 1.8 copies/µl (10 ag). The 1.8 copies/µl standard solution was freshly made before each experiment, by a 10-fold dilution of the standard containing 18 copies/µl.

## Results

In an attempt to further increase the sensitivity of virus-specific real-time PCR, we established a method called Amp-PCR where a random unbiased isothermal Phi29 amplification is combined with a virus-specific real-time PCR, the two of them performed in sequel in one tube (Figure 1A). To achieve this, we separated the two reactions physically by melting a gem of AmpliWax® in-between the two layers, so that the PCR master mix was locked in the bottom of the tube and separated from the Phi29 amplification reaction that was placed on top of the AmpliWax®-seal.

To test if the AmpliWax®-seal would interfere with the PCR reaction, we took 1 µl of purified JCV (∼1800 copies/µl) from a CSF clinical sample and added to a JCV-specific real-time PCR master mix. A pellet of AmpliWax® was melted on top and the PCR performed (Figure 1B). We saw no difference in PCR-performance with or without AmpliWax®, and therefore concluded that the AmpliWax®-seal did not interfere with the virus-specific real-time PCR.

**Figure 2:**
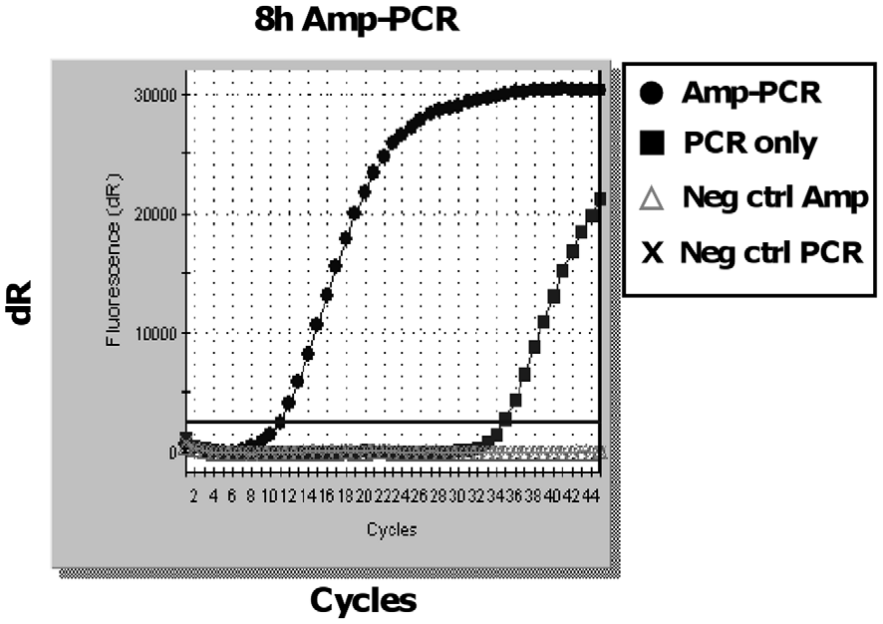
Amp-PCR on purified JCV from a clinical sample. Using purified viral JCV-DNA (∼36 copies/µl) from a clinical CSF sample, 8 hours of Amp-PCR was compared to PCR only. The ΔCt-value  = 24 demonstrating that Amp-PCR gives a strong amplification of the JVC material before the specific PCR. Negative control for both amplification and PCR is water.

To find a good balance between the volumes of the Repli-g reaction and the real-time PCR reaction in the Amp-PCR, we tested different ratios (1∶2, 1∶3, 1∶4 and 1∶5) (data not shown). The Repli-g reaction volume had to be large enough to allow for an acceptable sample volume to be amplified and at the same time not to be inhibitory for the real-time PCR. We tested 10 µl of Repli-g reaction which allowed for 1 µl of sample to be amplified. This volume was combined with 4 different real-time PCR reaction volumes: 20 µl (1∶2), 30 µl (1∶3), 40 µl (1∶4) and 50 µl (1∶5). Both 1∶3 and 1∶4 ratios worked well, while 1∶2 seemed inhibited and with 1∶5 we felt that the volume of PCR reagents was unnecessary large (data not shown). Based on this, for the further analysis we decided to use the ratio of 1∶3, with 10 µl of Repli-g reaction and 30 µl of PCR reaction, and performed Amp-PCR by combining 8 hours of Repli-g reaction with an AmpliWax®-sealed JCV-specific real-time PCR reaction (Figure 2). The sample used was 50-fold diluted purified JCV (∼36 copies/µl) from a CSF clinical sample. In parallel, as a control, the sample in just water was added on top of a wax-sealed PCR master mix (PCR only). The Amp-PCR amplified the JCV 16.8×10^6^-fold (ΔCt = 24) as compared to the sample that only went through the PCR-step (Figure 2). Furthermore, the negative control for the amplification demonstrates that high-molecular weight DNA normally produced in a Repli-g reaction in the absence of template (due to random extension of primer-dimers (Repli-g Midi kit handbook, Qiagen)), do not interfere with the specific real-time PCR reaction and is negative in the assay.

**Figure 3:**
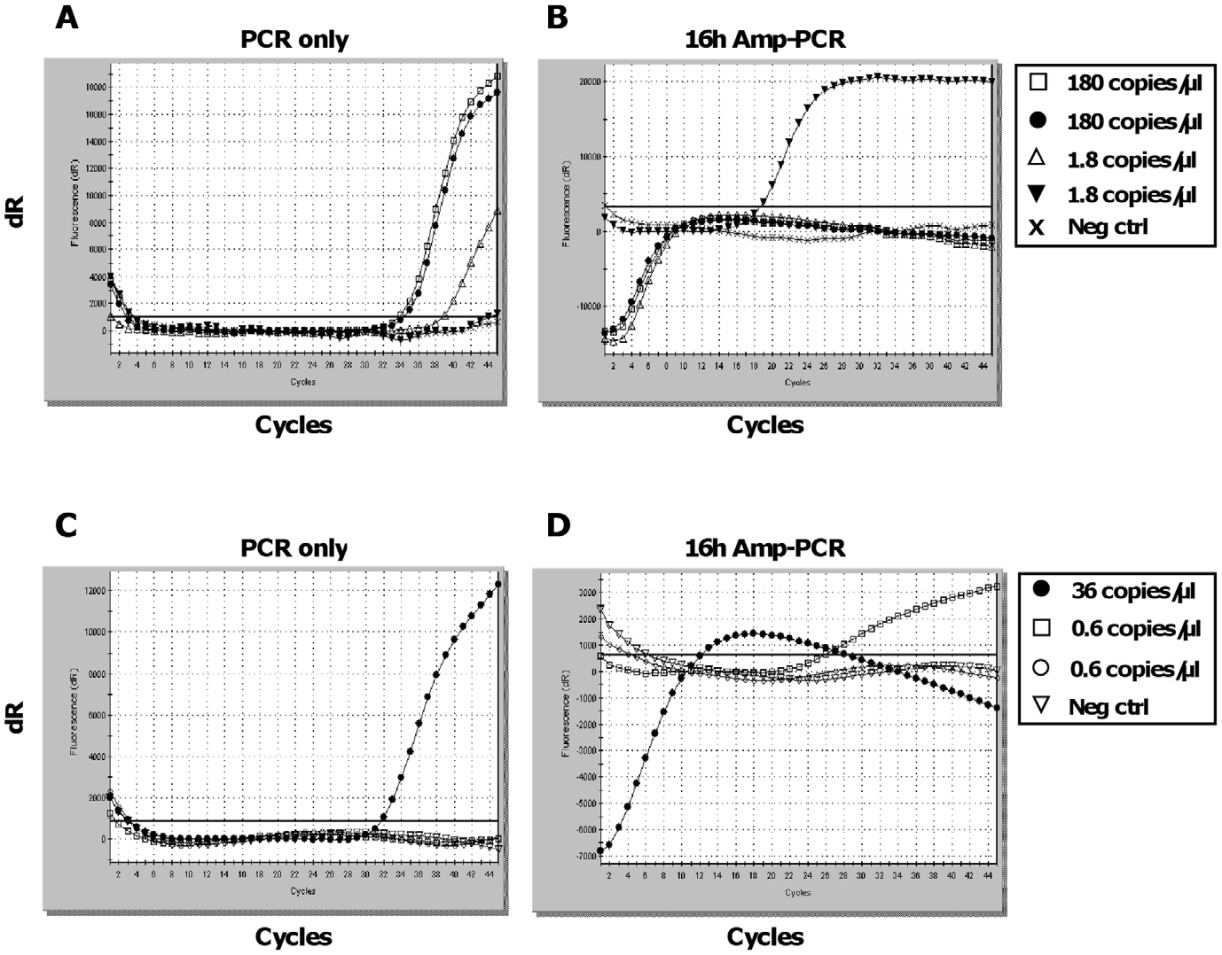
Sensitivity of the Amp-PCR method. To test the sensitivity of 16 hours Amp-PCR, we used two JCV-standard solutions (containing 180 and 1.8 copies/µl, respectively) and two dilutions of a JCV DNA from a clinical sample (containing 36 and 0.6 copies/µl, respectively). Sixteen hours of Repli-g reaction was followed by a JCV-specific real-time PCR reaction. (A) PCR only on duplicate samples of 180 and 1.8 copies/µl JCV-standards. Negative control for the PCR is water. (B) Amp-PCR on duplicate samples of 180 and 1.8 copies/µl JCV-standards (showing typical wave-shaped curves reported for over-saturated PCR-reactions). Negative control for the amplification is water. (C) PCR only on two diluted JCV DNA samples, containing 36 copies/µl and 0.6 copies/µl, respectively. Negative control for the PCR is water. (D) Amp-PCR on two diluted JCV DNA samples, containing 36 copies/µl (showing a typical wave-shaped curve reported for over-saturated PCR-reactions) and 0.6 copies/µl, respectively. Negative control for the amplification is water.

To test the sensitivity of the Amp-PCR method, we used the JCV Mad-1 pM1TC standard [11] at 180 and 1.8 copies/µl (1 fg and 10 ag of DNA, respectively) in duplicate samples (Figure 3A–B and Figure S1). We performed 16 hours of Repli-g reaction combined with the JCV-specific PCR. Both duplicates of 180 copies/µl were detected in the PCR only (Figure 3A) and both were strongly amplified after Amp-PCR (Figure 3B), with ΔCt =  ∼30 corresponding to a ∼1000×10^6^-fold increase in signal. Both duplicates showed a typical wave-shaped curve reported to be seen when excess sample is over-saturating the PCR reaction [12]. A multicomponent view of the wave-shaped curves confirmed typical sigmoid curves that were identical in shape to non-saturated samples, and were interpreted as JCV-specific results with Ct-values of ∼3-4 (Figure S1). The duplicate samples containing 1.8 copies/µl were clearly at the detection limit of the PCR reaction, with Ct-values of 39 and 44 (Figure 3A). However, both duplicates were strongly amplified (Ct = 17 and Ct =  ∼4) (Figure 3B) corresponding to between a 4×10^6^-fold (ΔCt  = 39−17 = 22) and 1×10^12^-fold (ΔCt  = 44−4 = 40) increase in signal, thereby showing the potential of the Amp-PCR method when analysing low copy number samples.

To investigate the reproducibility of the Amp-PCR assay, we did 10 parallel reactions using the JCV Mad-1 pM1TC standard [11] at 180 copies/µl (1 fg of DNA) (Table 1 and Figure S2). Ten PCR only reactions showed a mean Ct-value of 34.2±0.5 and 10 Amp-PCR reactions a mean Ct-value of 3.9±0.7, demonstrating good reproducibility. This corresponded to a 1300×10^6^-fold increase in signal. The Amp-PCR reactions showed the wave-shaped curves discussed above, but a multicomponent view confirmed typical sigmoid curves with Ct-values of 3-5 (Figure S2).

**Table 1:** Reproducibility of the Amp-PCR method.

Assay	I	II	III	IV	V	VI	VII	VIII	IX	X	Mean	Neg ctrl
**Amp-PCR** a	4	3	5	4	4	3	4	4	3	5	3.9±0.7	No Ct
**PCR only** b	33.4	33.9	33.8	34.0	34.3	34.8	34.5	34.7	34.0	34.8	34.2±0.5	No Ct

a Ten parallel Amp-PCR reactions using a JCV-standard containing 180 copies/µl.

b Ten parallel PCR only-reactions using a JCV-standard containing 180 copies/µl.

To test the sensitivity of Amp-PCR on a clinical sample, we used purified JCV DNA from a CSF clinical sample that was diluted 100-fold (∼36 copies/µl) and 6400-fold (∼0.6 copies/µl) (Figure 3C and D). The sample containing 36 copies/µl was detected in PCR only (Figure 3C) and strongly amplified after 16 hours Amp-PCR with a very strong JCV-signal (Ct =  ∼4), showing a typical wave-shaped curve reported for over-saturated PCR-reactions [12] and resulting in a 270×10^6^-fold increase in signal (ΔCt = 28). Neither of the duplicate samples containing 0.6 copies/µl gave a signal in PCR only (Figure 3C). However, one of the duplicates was amplified in 16 hours Amp-PCR (Ct = 27), while the other one was not (Figure 3D) demonstrating that samples under, or at, the detection limit of the real-time PCR assay may get amplified and show a good signal in our Amp-PCR-assay.

**Figure 4:**
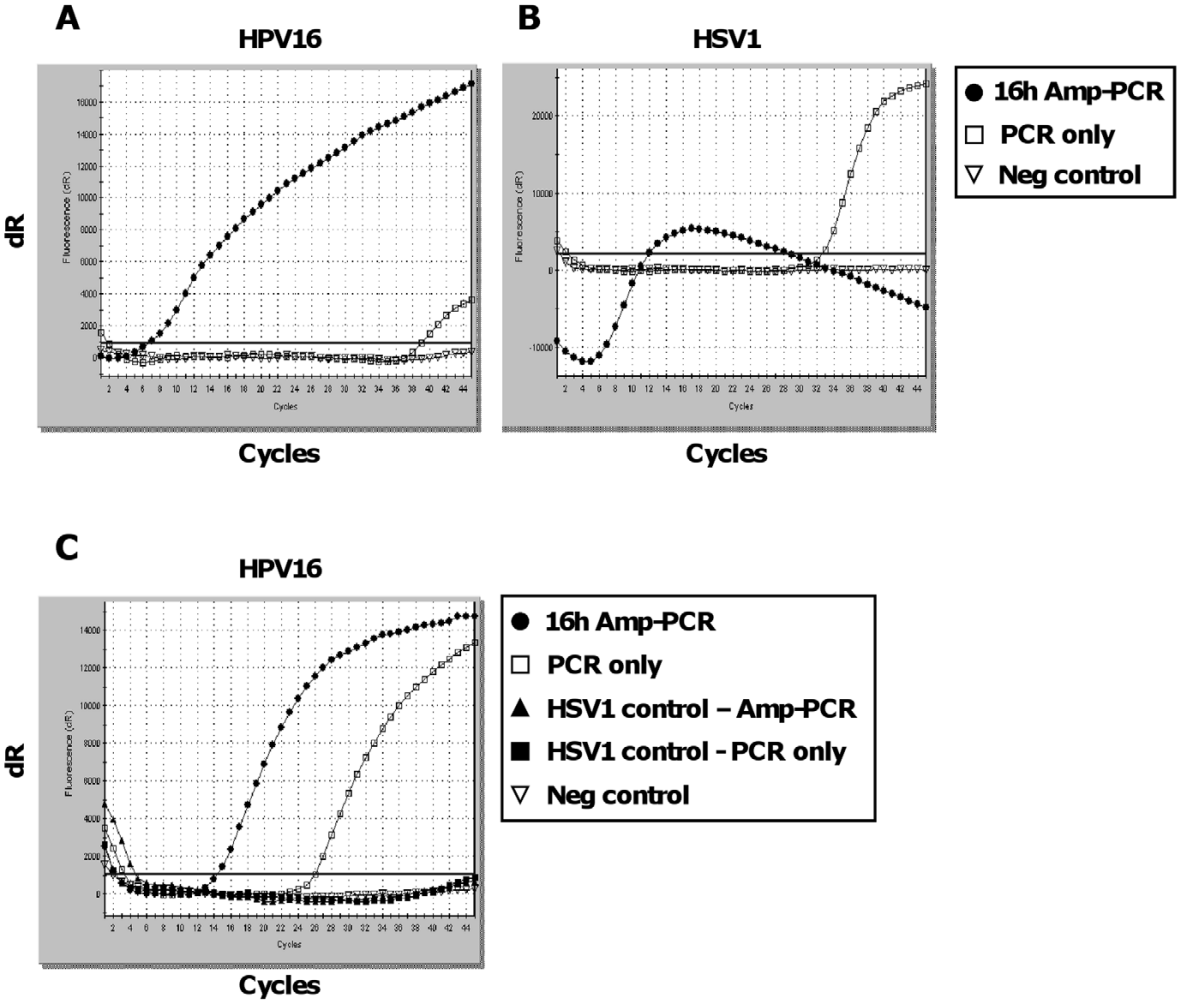
Testing Amp-PCR on other clinical samples. Amp-PCR was performed on a circular and a linear dsDNA virus purified from clinical samples, combining 16 hours of Repli-g with virus-specific real-time PCR. (A) Amp-PCR on purified HPV16. Negative control is water. (B) Amp-PCR on purified HSV1 (showing a typical wave-shaped curve reported for over-saturated PCR-reactions). Negative control is water. (C) HSV1-positive sample used as a negative control in a HPV16-specific Amp-PCR.

To show that this method could work equally well with other viral DNA samples, we tested 16 hours Amp-PCR on two additional clinical samples containing the DNA viruses HPV16 and HSV1, respectively (Figure 4). Amp-PCR on purified HPV16 from a cervical smear sample resulted in a 4300×10^6^-fold increase in signal (ΔCt = 32) compared to PCR only (Figure 4A). Amp-PCR on purified HSV1 from a clinical swab sample, showing a typical wave-shaped curve reported for over-saturated PCR-reactions [12], resulted in a 134×10^6^-fold increase in signal (ΔCt = 27) compared to PCR only (Figure 4B). To further demonstrate the specificity of the Amp-PCR, the HSV1-positive sample used in Figure 4B was included as a negative clinical control sample in a HPV16-specific Amp-PCR (Figure 4C). It was negative both before (PCR only) and after amplification (Amp-PCR), demonstrating no cross contamination and no un-specific PCR results between specimens.

## Discussion

A detection limit with maintained reproducibility of 10 copies per PCR reaction, have been reported for a JCV-specific real-time PCR [11]. With the additional use of a pre-amplification before the specific PCR, a single copy of DNA should theoretically, be detected with high reproducibility. In Figure 3B we show that 1.8 copy of JCV added to an Amp-PCR reaction was efficiently amplified and detected. Furthermore, in Figure 3D we show that diluted JCV containing 0.6 copy was detected by Amp-PCR but not by PCR only.

Nested PCR is used to increase sensitivity in PCR, but at the same time poses a severe risk of contamination in a clinical laboratory since amplified material from the first PCR-reaction is transferred to the second. Moreover, the need for two PCR reactions makes it more time consuming. The Amp-PCR method has eliminated this risk of cross-contamination since no transfer of pre-amplified material is needed. Since the pre-amplification and the specific PCR-reaction are run in the same tube, less time is spent on pipetting, making the method less labour intense and can be run overnight to fit into routine schedules. We show the Amp-PCR method to increase the signal of a specific PCR several million-fold (from 4×10^6^ to 1×10^12^) by increasing the amount of sample going into the PCR-reaction. Furthermore, the Phi29 DNA polymerase generates long products suitable for efficient PCR and is unbiased. Taken together, the Amp-PCR method appears to be a reliable and reproducible method that is capable of detecting amounts of sample normally under the detection limit of a specific PCR.

It could also be possible to combine the Amp-PCR method with a suitable reverse transcription (RT)-step, thereby enabling pre-amplification plus specific real-time PCR of mRNA to look at expression patterns or detect RNA viruses. This would help in the monitoring of low RNA viral loads seen in situations as with anti-retroviral treated patients or elite controllers during HIV infections. Furthermore, the pre-amplification could also be combined with a specific multiplex real-time PCR reaction, instead of a single-plex reaction as used here.

When analysing clinical samples, the sample volume used for purification of viral DNA, can easily be increased to more than 200 µl used here, thereby increasing the possibility of detection when working with low copy numbers. Furthermore, we found that removal of contaminating human genomic DNA by DNase-treatment during the purification step increased the sensitivity of the assay, since the Phi29 DNA polymerase amplifies any DNA present in the assay. The viral DNA is believed to be protected from DNase-degradation inside the viral particle during this step [13].

The Repli-g reaction contains random primers and Phi29 DNA polymerase which by MDA amplifies the DNA present in the sample. According to the Repli-g Midi protocol, the reaction should be run for 16 hours to reach maximum yield of amplified DNA (∼40 µg in 50 µl when starting with 10 ng, Qiagen). In our case, a reaction time of 16 hours ensures that all random primers are exhausted to reduce the risk of interference in the specific PCR that follows. We found that samples with high copy number of viral genomes exhausted the reagents in less than 16 hours. When samples with low copy number are used, the full reaction time of 16 hours is needed for the reaction to run out of reagents or to reach a point where the level of non-used random primers is low enough to not interfere with the downstream real-time PCR. High-copy samples generated an excess of products during the Repli-g reaction, demonstrating the capacity of the Phi29 polymerase to produce large amounts of material, which resulted in wave-shaped curves after real-time PCR (Figure 3B, 3D and 4). If the pre-amplification had been run in a separate tube, as with nested-PCR, one would only transfer minute amounts of the Repli-g product to the PCR master mix to not over-saturate the reaction. But since the reactions now appear in the same tube, all generated Repli-g product is transferred to the PCR master mix after the wax is melted. However, these curves are easily distinguished as positive samples demonstrating typical sigmoid curves with very low Ct-values (Figure S1 and S2). They are not false positive and should not be interpreted as false negative, and they only occur when high-copy number samples are analysed. Already at sample concentrations of around 36 copies/µl of JCV, the Amp-PCR can be over-saturated if being run for 16 h (Figure 3D), while a shorter incubation time will generate normal curves as seen in Figure 2. However, circular DNA, such as JCV, has been reported to be efficiently amplified by Phi29 polymerase through rolling circle amplification (RCA) [14] and would probably generate over-saturated reactions at a lower copy-number than seen for linear DNA samples. This assay was primarily developed to solve a re-occurring diagnostic problem with low-copy number samples.

The pre-amplification used here is a random isothermal MDA-reaction performed by the Phi29 DNA polymerase together with random hexamer primers, but other isothermal amplification-reactions, e.g. primase-based WGA (pWGA; Rapisome, BioHelix Corp.) could also be suitable. The Phi29 DNA polymerase does not efficiently amplify DNA fragments of ≤2 kb in size [15] (Repli-g Mini/Midi handbook, Qiagen), but works well on DNA of any origin from both non-cellular (virus) and cellular sources (bacteria, archae, eukaryotes), and on cDNA of sizes ≥2 kb. Since the pre-amplification is not specific, any suitable DNA present in the Repli-g-reaction will be amplified.

Thus, in clinical cases where the detection limit of a routine real-time PCR assay is not sensitive enough, an unbiased pre-amplification could be helpful. This could be when the virus is present at very low amounts and where detection early in a disease progression would benefit the patient with a better prognostic outcome. This is true for cases of suspected progressive multifocal leukoencephalopathy (PML) in the central nervous system in immunosuppressed patients [16], where an early correct detection of JCV viral DNA in the spinal fluid increases the chances for survival [17], [18]. Even if the real-time PCR used for a specific virus is very sensitive, it is always a risk for false negative results when analysing samples that are at the detection limit of the assay. Again, an unbiased pre-amplification could be helpful to bring up the copy number before the virus-specific real-time PCR assay. In situations where the sample is precious and irretrievable, a pre-amplification before a specific PCR would be helpful to reduce the amount of sample needed for the analysis. This could be dried blood samples on filter paper, or when working with ancient samples, forensic samples [19] or single-cells from culture or cell-sorting.

## Supporting Information

Figure S1 **Over-saturated samples in Figure 3 demonstrate typical sigmoid curves.** Multicomponent view of over-saturated curves from Figure 3, demonstrating typical sigmoid curves with a Ct-value of 3–4.
(PPTX)

Figure S2 **Over-saturated samples in Table 1 demonstrate typical sigmoid curves.** Multicomponent view of over-saturated curves used in Table 1, demonstrating typical sigmoid curves with a Ct-value of 3–5. (A) dR view of results after 16 h Amp-PCR using a JCV standard containing 180 copies/µl. (B) Multicomponent view of results after 16 h Amp-PCR using a JCV standard containing 180 copies/µl. (C) dR view of results after ‘PCR only’ using a JCV standard containing 180 copies/µl. (D) Multicomponent view of results after ‘PCR only’ using a JCV standard containing 180 copies/µl.
(PPTX)
