# Supplementary figures and images for: Amp-PCR: Combining a Random Unbiased Phi29-Amplification with a Specific Real-Time PCR, Performed in One Tube to Increase PCR Sensitivity
Source: PLoS One. 2010 Dec 31;5(12):e15719. doi: 10.1371/journal.pone.0015719 (PMC3013126; doi:10.1371/journal.pone.0015719)

## Slide 1
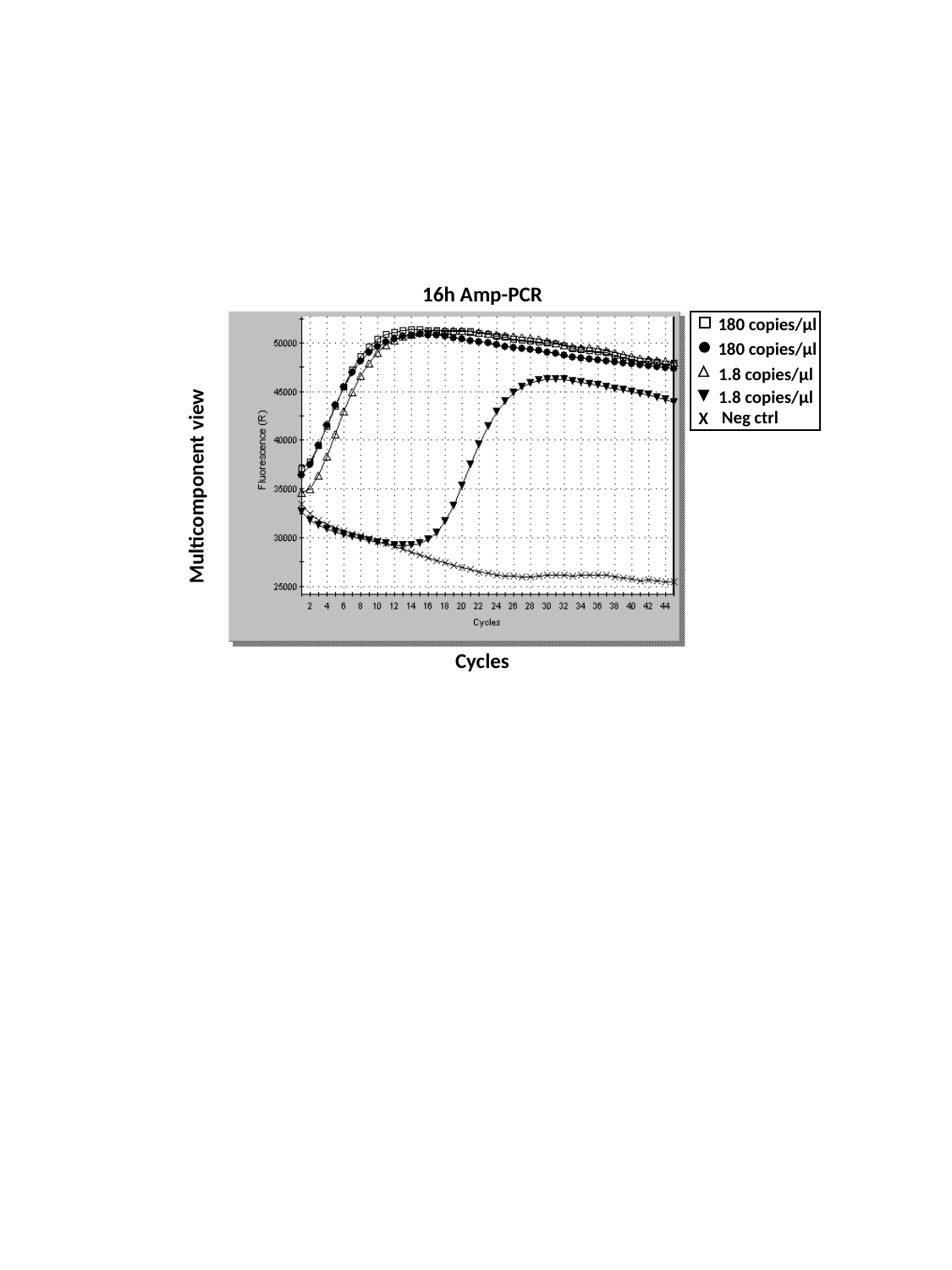

16h Amp-PCR
180 copies/µl
180 copies/µl
1.8 copies/µl
1.8 copies/µl
Neg ctrl
X
Multicomponent view
Cycles

Supplement: Figure S1 — Over-saturated samples in Figure 3 demonstrate typical sigmoid curves. Multicomponent view of over-saturated curves from Figure 3, demonstrating typical sigmoid curves with a Ct-value of 3–4. (PPTX) [file pone.0015719.s001.pptx]
