# Supplementary material for: Amp-PCR: Combining a Random Unbiased Phi29-Amplification with a Specific Real-Time PCR, Performed in One Tube to Increase PCR Sensitivity
Source: PLoS One. 2010 Dec 31;5(12):e15719. doi: 10.1371/journal.pone.0015719 (PMC3013126; doi:10.1371/journal.pone.0015719)
Supplement: Figure S2 — Over-saturated samples in Table 1 demonstrate typical sigmoid curves. Multicomponent view of over-saturated curves used in Table 1, demonstrating typical sigmoid curves with a Ct-value of 3–5. (A) dR view of results after 16 h Amp-PCR using a JCV standard containing 180 copies/µl. (B) Multicomponent view of results after 16 h Amp-PCR using a JCV standard containing 180 copies/µl. (C) dR view of results after ‘PCR only’ using a JCV standard containing 180 copies/µl. (D) Multicomponent view of results after ‘PCR only’ using a JCV standard containing 180 copies/µl. (PPTX) [file pone.0015719.s002.pptx]

## Slide 1
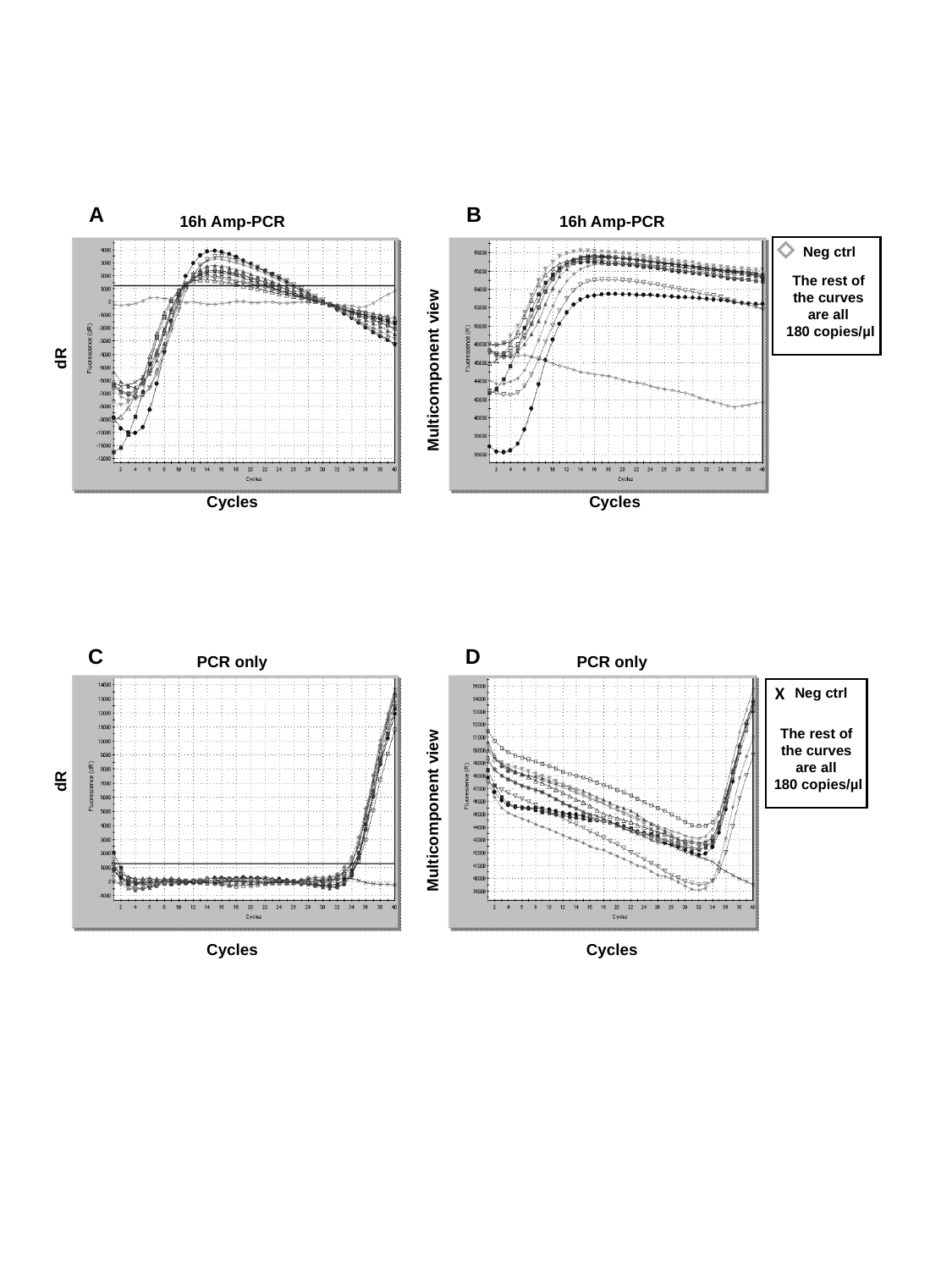

A
B
16h Amp-PCR
16h Amp-PCR
Neg ctrl
The rest of
the curves
are all
180 copies/µl
dR
Multicomponent view
Cycles
Cycles
C
D
PCR only
PCR only
X
Neg ctrl
The rest of
the curves
are all
180 copies/µl
dR
Multicomponent view
Cycles
Cycles
